# Strain Engineering in Graphene at the Nanometer Scale

**DOI:** 10.1021/acs.nanolett.5c03926

**Published:** 2025-10-30

**Authors:** Paula García-Mochales, Antonio J. Martínez-Galera

**Affiliations:** † Departamento de Física de Materiales, 16722 Universidad Autónoma de Madrid, Madrid E-28049, Spain; ‡ Condensed Matter Physics Center (IFIMAC), Universidad Autónoma de Madrid, Madrid E-28049, Spain; § Instituto Nicolás Cabrera, Universidad Autónoma de Madrid, Madrid E-28049, Spain

**Keywords:** Straintronics, 2D materials, Nanomanipulation, Scanning tunneling microscopy, Nanoparticles

## Abstract

The ability to induce and characterize strain in the atomic lattice of 2D materials, localized within only a few nanometers around specific positions, is a major challenge for the development of straintronics. In this work, the interaction between Si nanoparticles and the surface of graphene/Ru(0001) is employed to induce local strain in the latter. The strain field has been mapped at the nanoscale by scanning tunneling microscopy (STM), using the moiré pattern intrinsic to graphene/Ru(0001) surfaces as a magnifying lens. The induced strain is found to be confined within only a few nanometers around each nanoparticle. To achieve more accurate control, strain engineering at the nanometer scale was successfully performed by manipulating nanoparticles through the STM tip. This approach to controlled strain could provide a key tool for exploring new physics arising from strain in 2D materials.

## Graphical abstract

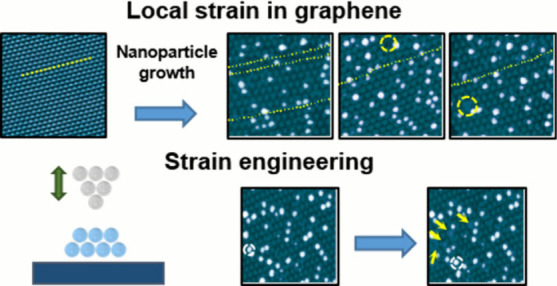

Graphene, with a tensile strength of 130 GPa and a Young’s modulus of 1 TPa, is the strongest material currently available.^1^ These unprecedented mechanical properties arise from the strength of its C–C bonds, which make this 2D material capable of sustaining reversible tensile strain of almost 30%.^1^ This feature is of great relevance, as the introduction of strain involves variations in the bonding distances and angles, giving rise to changes in the size and the symmetry of the atomic lattice of this material, and consequently in its electronic, vibrational, magnetic, optical, and topological properties.^2–14^

Achieving accurate control over these properties by inducing controlled strain, which is the focus of the research area known as straintronics, is currently one of the major challenges within the 2D materials community.^15–21^ However, while the effects of strain engineering on the properties of graphene have captivated considerable interest for more than a decade,^2–4,6–8,12,22^ research aimed at developing accurate engineering approaches has lagged behind,^23^ and precise control over lattice deformation at the local scale, in the range of a few nanometers, is still needed. In this regard, the development of such approaches will be the key to fully developing the promising potential of straintronics, allowing well-controlled and accurate experiments to study the emerging properties arising from strain.

Between the different approaches for strain engineering, a widely used one is to place graphene onto a flexible substrate and straining the latter by either stretching or bending.^7,24–29^ In a more sophisticated variant, graphene is positioned onto a piezoelectric material, which is then deformed by applying an electric field.^30,31^ These techniques have as a main drawback a lack of local selectivity (nonlocalized strain) along with effects such as sliding of graphene over the support. Among the approaches with local precision, it is important to mention the nanoindentation with an AFM tip on a graphene sheet placed onto a patterned substrate.^1,32,33^ While this method has provided valuable information about the mechanical properties of graphene,^1^ it is unsuitable for investigating the influence of strain on some specific local properties. The origin of this disadvantage resides in the fact that the tip is placed over the strained area, making it very difficult to obtain an image of the strain field with nanometric precision. On top of all the above-mentioned drawbacks, some of these methods produce homogeneous strain. However, achieving inhomogeneous strain to generate regions with varying electronic, optical, and vibrational properties within the same sample would imply a key step in the development of straintronics, allowing one to investigate a wide phenomenological variety and to combine different functionalities on the same nanomaterial.

In this work, we provide a novel approach for strain engineering of graphene, allowing us to achieve both an inhomogeneous localized deformation within a few nanometers around specific positions and hence an inhomogeneous strain, as well as direct mapping and quantification of the strained region. Specifically, the growth of Si nanoparticles on graphene/Ru(0001) surfaces is found to change the bonding landscape at the interface between the 2D material and the metal support, producing local deformations. By using the moiré pattern characteristic of graphene/Ru(0001) surfaces as a magnifying lens, precise mapping of the strain field is achieved by employing scanning tunneling microscopy (STM). Pursuing a more precise engineering, the strain field has been nanomanipulated by the controlled removal of individual nanoparticles with the STM tip. The findings of these experiments suggest the formation of a network of stress fields, each associated with a single nanoparticle, and stress transfer along the graphene layer, which behaves as an elastic membrane.

Experiments were performed in a home-built ultrahigh-vacuum (UHV) system, equipped with a variable-temperature scanning tunneling microscope (VT-STM) and a low-energy electron diffraction (LEED) optics for sample characterization.^34^

Ru­(0001) surfaces were cleaned by repeated cycles of Xe^+^ ion sputtering at 900 eV followed by annealing at 850 °C, first at a partial pressure of 2 × 10^–6^ Torr of O_2_ and then without oxygen supply. Subsequently, the sample was flash annealed at 1100 °C in the absence of any oxygen partial pressure supplied to the chamber. Single-layer graphene was grown by chemical vapor deposition at 850 °C using ethylene as a precursor at a partial pressure of 3 × 10^–7^ Torr, which was kept during 300 s.

Si was deposited from a current-heated wafer made from this element. The deposition rate was calibrated against the current flowing through the wafer by depositing Si over freshly prepared Ru(0001) surfaces and performing a posterior STM analysis of the coverage.

STM data acquisition and analysis were performed by using the WSxM software.^35^ For STM imaging, the bias voltage *V*_*S*_ was applied to the sample, while the tip was connected to the ground. STM data acquisition, including nanoparticle nanomanipulation, was conducted at room temperature (RT).

The apparent height histograms that are shown afterwards were generated from STM images using a custom computational code developed specifically for this purpose.^36^ The code measures the apparent height of the highest point of each nanoparticle relative to the minimum height measured in a nearby empty moiré cell.

As a reference, Figures 1a and b summarize the main structural properties of the graphene/Ru(0001) surfaces obtained in this work following the procedure described above. In detail, Figure 1a shows an STM topograph displaying the moiré superstructure characteristic of graphene/Ru(0001) surfaces.^37–39^ As observed, the pattern follows a hexagonal lattice (see the black rhombus) of high structural perfection. In this regard, the histogram in the inset developed from STM data obtained on different samples represents the deviations found for moiré crests with respect to their expected positions in a perfect moiré superlattice. The reduced number of counts in the histogram is a consequence of the low probability of finding measurable distortions in the moiré pattern for the pristine graphene/Ru(0001) surfaces obtained in the present work. Accordingly, the LEED pattern displayed in Figure 1b shows, together with the diffraction spots associated with the atomic lattices of graphene and of the Ru(0001) surface, these related to the periodicity of the moiré superstructure resulting from their superposition. To better interpret the different diffraction features, Figure 1c shows a schematic illustration of the LEED pattern provided in Figure 1b.

**Figure 1:**
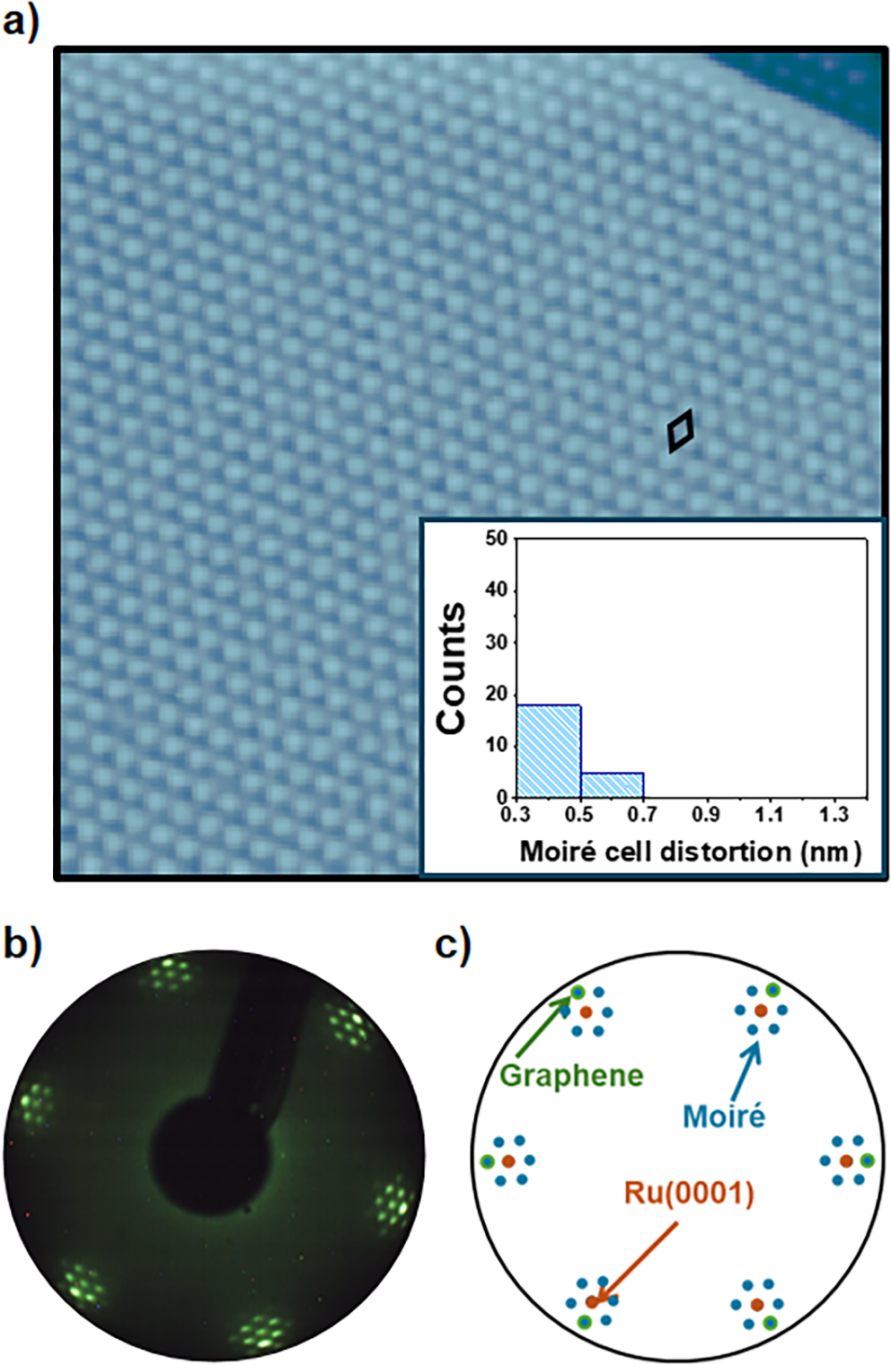
Structural characterization of graphene/Ru(0001). **a)** STM image illustrating the high structural perfection of the graphene/Ru(0001) surfaces grown in this work. The histogram in the inset represents deviations of the moiré crests from their expected positions in a perfect moiré superlattice. This histogram was developed from a number of STM images acquired over different pristine graphene/Ru(0001) surfaces. **b)** Typical LEED pattern acquired on graphene/Ru(0001). Electron energy: 75 eV. **c)** Schematic representation illustrating the origin of the different diffraction features observed in b). Tunneling parameters: a) *V* *_S_* = −2.73 V, *I*_*t*_ = 25 pA, size: 75 × 75 nm^2^.

Si exposure of the graphene/Ru(0001) surfaces yields the growth of atomic aggregates, which exhibit a certain tendency to be in registry with the moiré pattern (see Figure S1 in the Supporting Information). Figure 2a–c shows representative STM images acquired during the characterization of the spatial distribution of the nanoparticles as a function of the amount of Si deposited. It can be seen from the STM images that the higher the Si coverage, the more nanoparticles are present on the graphene/Ru(0001) surfaces. This effect is quantitatively confirmed by the evolution with coverage of the occupancy factor, which represents the average number of nanoparticles that exist per moiré unit cell (see Figure 2g). The fairly linear tendency of the occupancy factor at low Si coverages is in line with previous studies on the growth of nanoparticles on graphene/metal surfaces.^40^

**Figure 2:**
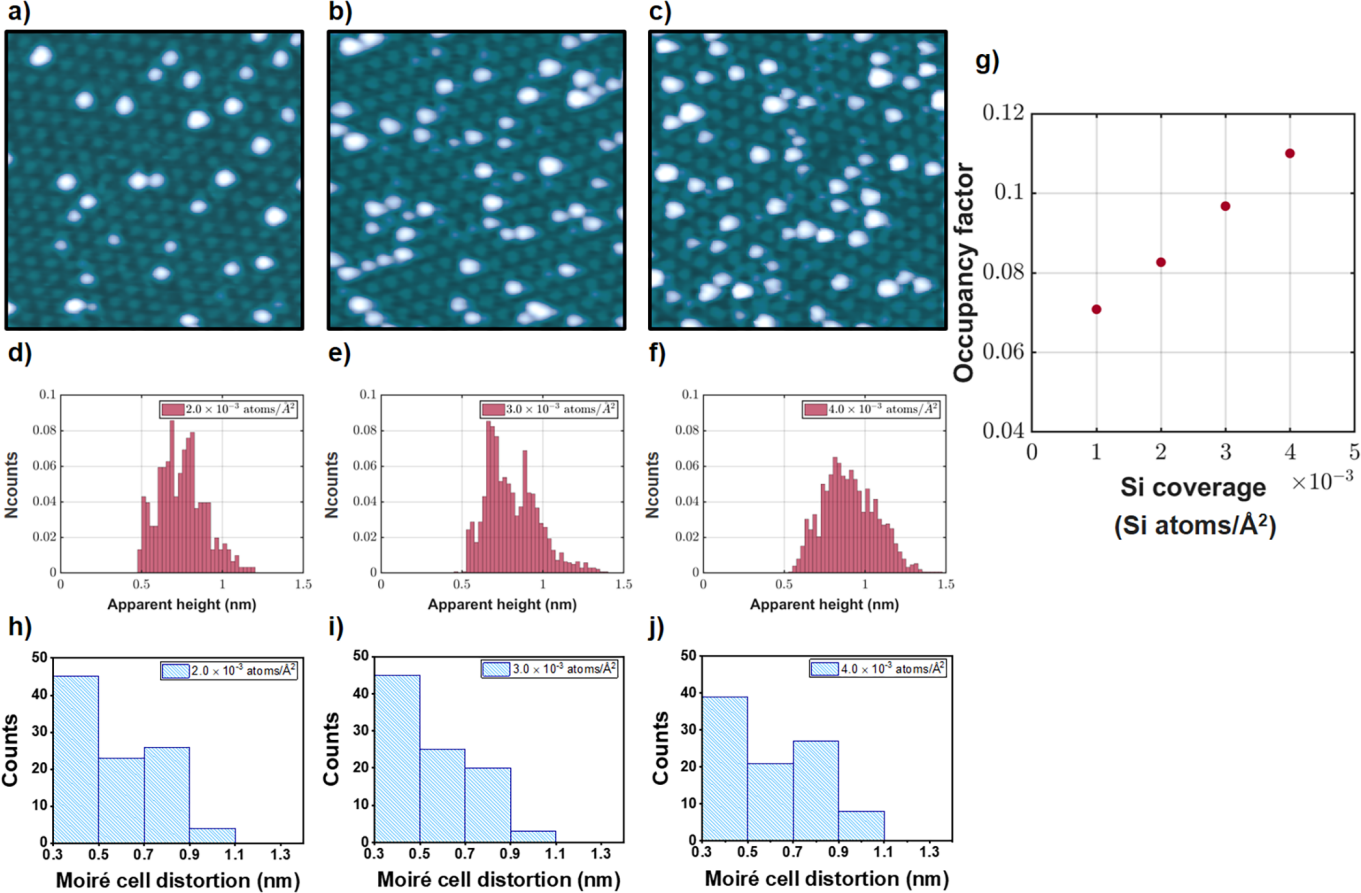
Growth and characterization of Si nanoparticles on graphene/Ru(0001). **a–c)** Sequence of STM images illustrating the evolution of the spatial distribution of the Si nanoparticles with increasing the amount of Si deposited over the graphene/Ru(0001) surface. Specifically, these images are representative of the respective STM measurements performed for Si coverages of 2 × 10^–3^, 3 × 10^–3^, and 4 × 10^–3^ atoms/Å^2^. **d–f)** Apparent height histograms, obtained from the analysis of STM images acquired respectively for Si coverages of 2 × 10^–3^, 3 × 10^–3^, and 4 × 10^–3^ atoms/Å^2^, illustrating the evolution of the nanoparticles with the amount of Si deposited over the graphene/Ru(0001) surface. *Ncounts* represents the number of nanoparticles found with apparent heights within each bin normalized to the total number of aggregates analyzed in each histogram. **g)** Evolution of the moiré occupancy factor with increasing amount of Si deposited over the graphene/Ru(0001) surface. The occupancy factor represents the average number of nanoparticles per moiré supercell. **h–j)** Histograms quantifying the strain in the moiré superstructure after nanoparticle growth for Si coverages of 2 × 10^–3^, 3 × 10^–3^, and 4 × 10^–3^ atoms/Å^2^, respectively. Tunneling parameters: a) *V*_*S*_ = −2.0 V, *I*_*t*_ = 20 pA, size: 50 × 50 nm^2^; b) *V*_*S*_ = −1.8 V, *I*_*t*_ = 9 pA, size: 50 × 50 nm^2^; c) *V*_*S*_ = −1.02 V, *I*_*t*_ = 17 pA, size: 50 × 50 nm^2^.

To extract insights into the internal structure of the nanoparticles, the apparent height distributions of the atomic aggregates have been studied as a function of the Si coverage (see Figure 2d–f). It can be observed how, in the coverage range analyzed, the histograms move toward slightly higher apparent heights, evidencing a slow increase in size of the nanoparticles as the amount of Si deposited on the graphene/Ru(0001) surfaces increases.

An interesting effect associated with the formation of the Si aggregates is the development of irregularities in the moiré pattern of the graphene/Ru(0001) surface. This effect is more obvious in the STM topograph displayed in Figure 2c, which exhibits the larger Si coverage. These moiré distortions are quantified by histograms, which are based on measurements of the deviation of the actual moiré crest positions from those expected in a perfect moiré superlattice (Figure 2h–j). As observed, the moiré deviations can reach up to 1 nm; however, larger distortions tend to be less frequent than smaller ones. Taking into account that the periodicity of the moiré superstructure is approximately 3 nm, a deviation of 1 nm corresponds to roughly 30%. Also, it can be noticed that while increasing the amount of Si deposited on the graphene/Ru(0001) surface leads to more distortions, there are no significant differences in the magnitude of these distortions for the various Si coverages, as evidenced by the comparison of the histograms.

These findings about the quantification of the distortions can be interpreted as follows: On one hand, increasing the amount of Si within the range of coverages analyzed leads to a higher number of nanoparticles, which in turn raises the frequency of encountering distortions. On the other hand, due to the 3D growth of the nanoparticles, an increase in Si coverage also results in a greater number of Si layers per nanoparticle. Then, the lack of a systematic increase in the magnitude of moiré distortions as the Si coverage becomes larger suggests that the distortions stem mainly from the interaction of Si atoms in the base layer of the nanoparticles with C atoms of the underlying graphene layer.

Now, the different types of strain in the moiré pattern found in this work are analyzed in more detail. The distortions most commonly found after nanoparticle growth consist of a significant bending of the high-symmetry directions of the moiré pattern (see yellow dotted traces in Figure 3a–c). This type of induced strain in the moiré superstructure becomes more clear by comparing the STM images acquired after the growth of the nanoparticles with the reference ones obtained on pristine graphene/Ru(0001) surfaces (compare with Figure 1a). As mentioned above, these irregularities tend to appear more frequently in the images obtained on samples with higher Si coverages that display higher occupancy factors (compare Figure 2a and c). It is important to mention that although irregularities have been observed in previous works also in nanoparticle-free graphene/Ru(0001) samples, they were found to be less extended over the surface.^39^

**Figure 3:**
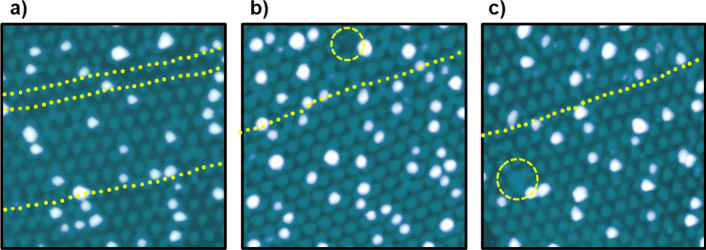
Qualitative analysis of the induced strain in graphene. **a)** STM image illustrating the bending of the high-symmetry directions of the moiré pattern after the growth of the Si nanoparticles. Yellow dotted traces are included to guide the eye, facilitating the identification of these deformations. **b)** STM image showing an alteration of the moiré apparent corrugation in the form of a missing protruding feature (see inside the circumference). **c)** STM image acquired over a region displaying an alteration in the moiré landscape consisting of an extended protruding feature (see inside the circumference). Tunneling parameters: a) *V*_*S*_ = −2.0 V, *I*_*t*_ = 50 pA, size: 45 × 45 nm^2^; b) *V*_*S*_ = −1.8 V, *I*_*t*_ = 0.2 nA, size: 45 × 45 nm^2^; c) *V*_*S*_ = −2.07 V, *I*_*t*_ = 16 pA, size: 45 × 45 nm^2^.

Besides the distortions mentioned above, another effect that has been also observed after the growth of the Si nanoparticles is the local modification of the apparent corrugation of the moiré pattern of graphene/Ru(0001). Two different variants of this effect have been found. On the one hand, the protruding feature within a moiré supercell is sometimes found to be missing, as shown in Figure 3b (see the interior of the region delimited by the yellow circumference). On the other hand, additional protrusions within moiré supercells are occasionally observed as well (see Figure 3c). As a consequence, the moiré apparent corrugation is no longer well-defined; rather, the STM detects a protruding feature often extending over adjacent moiré supercells, altering the pattern, as highlighted by the yellow circumference shown in Figure 3c.

The moiré deformations observed could have their origin in the establishment of bonds between Si atoms of the nanoparticles and C atoms of the graphene layer placed underneath. As a result of these bonds, the specific bonding landscape between the graphene layer and the underlying Ru(0001) surface, which is responsible for the geometry of the pristine moiré pattern, would be altered. Then, this modification of the interfacial chemistry could lead to additional stress in the graphene layer, the above-described irregularities being the strain resulting from its relaxation.

The moiré distortions observed after nanoparticle adsorption offer an unprecedented opportunity to explore the development of a route for precise strain engineering at the local level, based on the manipulation of nanoparticles. The methodology employed for nanoparticle manipulation is described in detail elsewhere.^41–44^ Here, this methodology will be described only briefly. Specifically, after a region of the sample is imaged, a specific nanoparticle is selected for removal. Then, the STM tip is positioned directly above it. With the tip in place, the feedback loop is disabled, and the tip is approached toward the targeted nanoparticle at a steady pace over a typical distance of approximately 0.6 nm. Then, the tip is retracted, and the feedback loop is restored. Figure 4 shows a succession of images acquired over a region in which several nanoparticles were sequentially removed by following this procedure. In each image, the nanoparticle that was to be removed, and therefore is not present in the following topograph, is surrounded in white. In contrast, the locations of the nanoparticles for which an unsuccessful removal attempt was made are encircled in red. Already after the extraction of the first nanoparticle, clear alterations in the moiré landscape began to appear (see yellow arrows in Figure 4b). They are found, respectively, in a moiré supercell adjacent to the removed nanoparticle, in a second and a fourth nearest neighbors, and in another one more than 24 nm away. Nevertheless, upon removal of the second nanoparticle, the respective interfacial alterations in the two supercells nearest to the initially extracted one were almost completely corrected, even though there were three other nanoparticles between the one removed in the second place and the positions where the moiré pattern was partially recovered (Figure 4c). The fourth nanoparticle manipulation attempt was unsuccessful, and the targeted Si aggregate changed its position instead of being removed (see the green circumference in Figure 4e). Here it is noteworthy that the region around all three nanoparticles that had been extracted up to that point began to look somehow defective (see Figure 4e). This effect becomes more obvious in the respective images acquired following the removal of the next two nanoparticles, in which the formation of a highly defective region is observed (see Figures 4f and g). It should be mentioned that the huge distortions observed in that region cannot be explained as the result of changes in the resolution, as outside that area the moiré pattern is observed clearly. Interestingly, after the extraction of other nanoparticle, all these distortions were corrected (see Figure 4h), and the moiré pattern was completely restored within that region. However, these distortions came back again after the acquisition of other image without performing any nanoparticle removal attempt (see Figure 4i). It suggests that the alterations observed cannot be attributed to a big deposit from the tip, as the whole distortion is repaired next to the nanomanipulation of the seventh nanoparticle (see Figure 4h), and it came back again after the subsequent acquisition of an image without removing any nanoparticle or observing any sign of a tip change (see Figure 4i). In this line, another indication to exclude a big deposit from the tip as the origin of the observed alterations in the moiré landscape is their gradual appearance exclusively within the region indicated by the yellow elliptical contour (see images of Figure 4e and f). Also, the possibility that the large distortion is an artifact caused by imaging with a multiple tip is unlikely. After acquisition of the image displayed in Figure 4f, where the huge distorted region is already present, the nanoparticle encircled in white in Figure 4f was successfully removed. As shown in Figure 4g, the targeted nanoparticle is indeed absent, yet the distorted region persists and retains the same shape. In this manipulation event, the tip apex has necessarily changed from one image to the other; however, the distorted region is still present. Finally, after the unsuccessful attempt to remove the nanoparticle encircled in red in Figure 4i, these local distortions were partially corrected again so that the moiré was almost completely restored (see Figure 4j).

**Figure 4:**
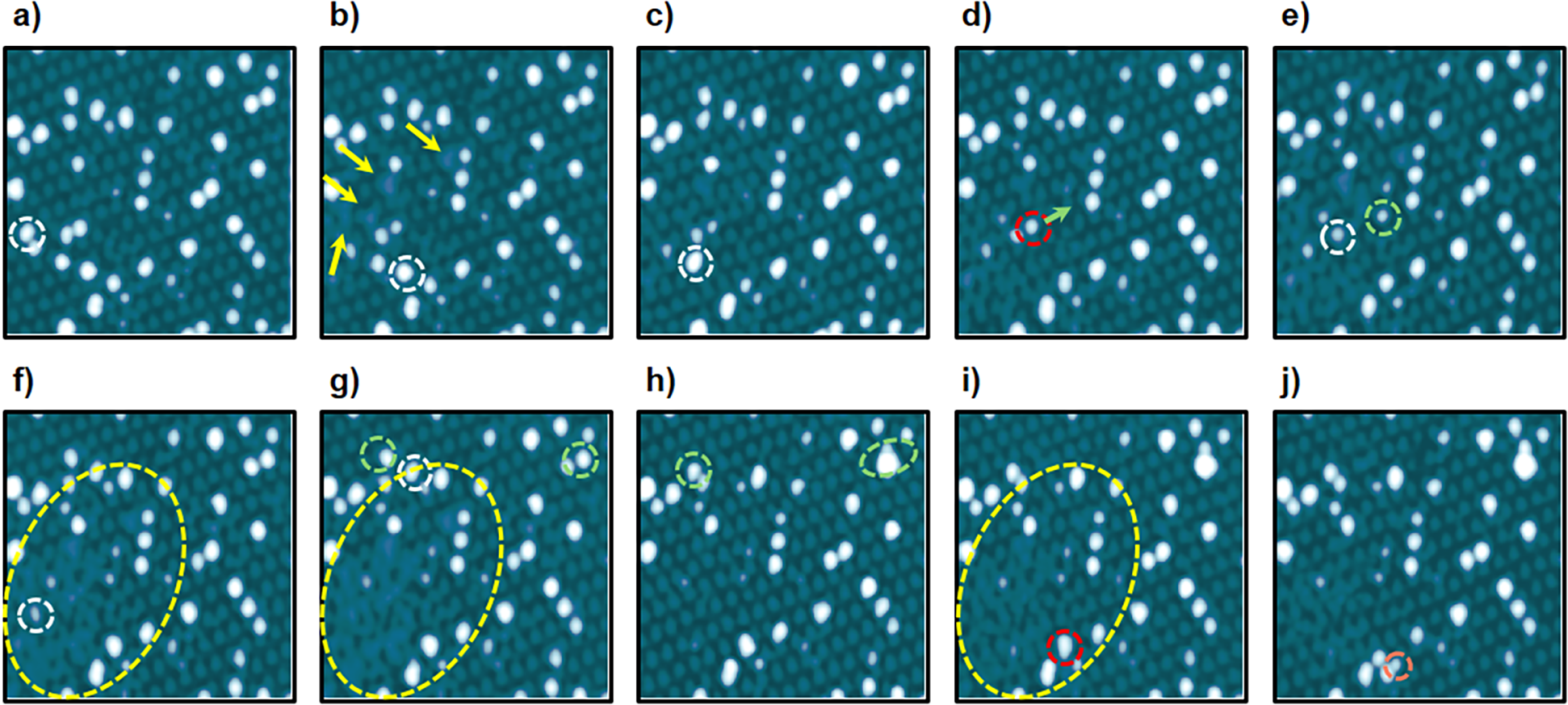
Strain engineering in graphene by nanomanipulation with the STM tip. **a**–**j)** Series of STM images acquired along sequential removal attempts of nanoparticles. The white circumference in each image indicates the nanoparticle targeted for removal in each step of the series, while red circumferences indicate unsuccessful removal attempts. Likewise, each green circumference shows the displacement of a nanoparticle following the nanomanipulation of other aggregates, while the elliptical contours highlight extended changes in the moiré geometry. The orange circumference indicates Si desegregation after a nanoparticle removal attempt. Tunneling parameters: *V*_*S*_ = −2.4 V, *I*_*t*_ = 15 pA, size: 50 × 50 nm^2^ for all images.

Besides moiré distortions, other effects observed during the nanomanipulation experiments include the displacement and splitting of the nanoparticles. In particular, after the extraction of the Si aggregate highlighted in Figure 4g with a white circumference, another one in its vicinity, which is encircled in green, is displaced to an adjacent moiré supercell (compare with Figure 4h). Additionally, in the course of that nanoparticle extraction event, another Si aggregate, which is encircled in green at the top-right part of the STM image displayed in Figure 4g, also changes its position (compare with Figure 4h). Here, it must be noted that this nanoparticle was positioned more than 27 nm away from the one removed. On the other hand, after the unsuccessful attempt to remove the last nanoparticle of the series (the one encircled in red in Figure 4i), there is some disaggregation of Si, which generates a new nanoparticle in an adjacent moiré supercell (see the orange contour in Figure 4j).

In the following, we provide an interpretation of the experimental findings presented in Figure 4. The changes in the moiré pattern observed after removal attempts of nanoparticles may be related to additional stress induced in the graphene layer after this nanomanipulation procedure. Nanoparticle removal involves modification of anchor points between the graphene layer and the Ru(0001) surface. It is also expected to occur if the removal attempt is unsuccessful but induces changes in the internal structure of the nanoparticles. In both cases stress generation at the interface is expected, forcing the system to move to a new state. It could involve changing the relative positions of the carbon atoms with respect to the ruthenium ones, thus generating the observed distortions. This evolution to a new state could imply going through different metastable states. This process could sometimes last on time scales of minutes, which would explain the observation of changes after a certain time without perturbing the sample (compare Figures 4h and i). Concerning the influence radius, the observed effects often go beyond the immediate vicinity of the nanoparticle extracted. For instance, in Figure 4b, a moiré distortion is observed at more than 24 nm from the removed nanoparticle. Additionally, a nanoparticle shifts its position after the removal of other aggregate located more than 27 nm away (see Figures 4g,h). It could imply that the Si aggregates generate a network of stress fields that interact with each other. It would give rise to stress propagation along the graphene layer, which behaves as an elastic membrane. In line with this argument, it is worth mentioning that the observed moiré distortions are unlikely to originate from Si intercalation at the interface between the graphene layer and the metal substrate. Although there is available literature reporting on the intercalation of different elements, including Si, at the graphene/Ru(0001) interface,^45–47^ in all these cases achieving that intercalation required a postannealing procedure after deposition of the element over the graphene/Ru(0001) surface. In our case, the experiments were performed on as-grown samples consisting of nanoparticles formed by Si deposition on graphene/Ru(0001) at RT, with no postgrowth annealing. One still could think that intercalation could be induced by nanomanipulation events. Nevertheless, in this case, it would be difficult to explain why the distorted region nearly disappears after a certain point.

In conclusion, the growth of Si nanoparticles is shown to induce local strain in graphene monolayers grown on Ru(0001) surfaces. The induced strain has been characterized by using the moiré pattern characteristic of that graphene–metal interface as a magnifying lens. This strain is found to be localized within only a few nanometers around each nanoparticle. The origin of this strain has been tantalizingly linked to changes in the interfacial bonding landscape of the graphene/Ru(0001) system after the formation of the Si nanoparticles. The induced strain was nanomanipulated by the controlled removal of nanoparticles with the STM tip. Nanoparticle removal is found to cause effects at distances of >20 nm from the site of nanomanipulation. It is suggested that nanoparticle adsorption creates a network of stress fields that interact with each other, leading to stress propagation along the graphene layer. The generation and nanomanipulation of strain demonstrated in this work constitute an approach providing ultimate control, which is expected to contribute significantly to developing straintronics. Specifically, the unprecedented local precision provided by this approach could imply the first step toward accurate studies of newly emergent properties arising by strain through measurements in strained and not strained surrounding regions.

## Supplementary Material





## Notes

Positioning of nanoparticles over the moiré pattern (PDF)
Video illustrating the phenomenology observed along sequential nanoparticle manipulation (AVI)

## △

Technische Universität München, Munich D-85748, Germany

## ▽

Ludwig-Maximilians-Universität, Munich D-80799, Germany.
