# Supplementary material for: Strain Engineering in Graphene at the Nanometer Scale
Source: Nano Lett. 2025 Oct 30;25(45):16097–103. doi: 10.1021/acs.nanolett.5c03926 (PMC12616772; doi:10.1021/acs.nanolett.5c03926)
Supplement: Supplementary file 1 [file nl5c03926_si_001.pdf]

Supplementary information

Strain engineering in graphene at the nanometer scale

Paula García-Mochales,[1,§,‡] *and Antonio J. Martínez-Galera*[1,2,3*]

[1]Departamento de Física de Materiales, Universidad Autónoma de Madrid, Madrid E-28049, Spain

[2]Condensed Matter Physics Center (IFIMAC), Universidad Autónoma de Madrid, Madrid E-28049, Spain

[3]Instituto Nicolás Cabrera, Universidad Autónoma de Madrid, Madrid E-28049, Spain

[§]Present address: Technische Universität München, Munich D-85748, Germany

[‡]Present address: Ludwig-Maximilians-Universität, Munich D-80799, Germany

* antonio.galera@uam.es

S1. Positioning of Si nanoparticles over the moiré pattern of graphene/Ru(0001)

Figure S1a shows a representative STM image obtained after the deposition of Si with a coverage of 2×10^{-3} atoms/Å² onto a graphene/Ru(0001) surface. Figure S2b shows the same image with a green lattice corresponding to the periodicity of the moiré pattern overlaid. It can be observed that the Si nanoparticles grown display a certain tendency to be placed in registry with the moiré pattern.

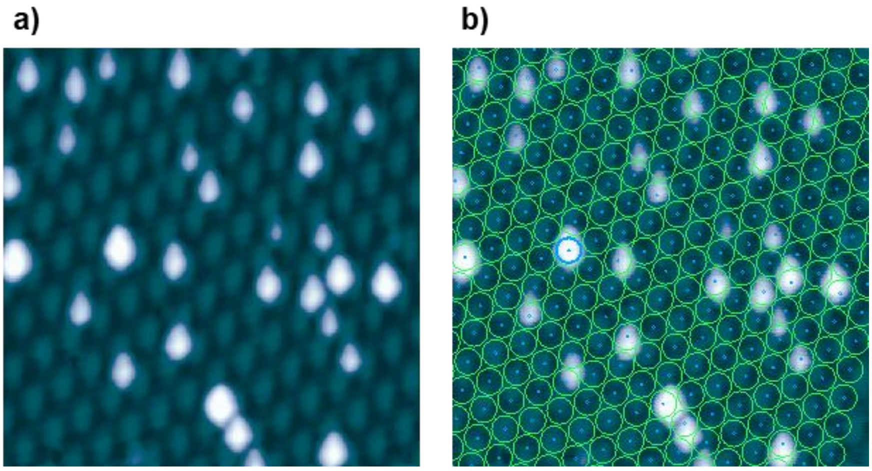

Figure S1. Positioning of the Si nanoparticles over the moiré pattern of graphene/Ru(0001). **a)** Representative STM image acquired after depositing 2×10^{-3} atoms/Å^2 of Si over a graphene/Ru(0001) surface. Tunneling parameters: $V_S = -1.8$ V, I_t = 9 pA, size: 45 × 45 nm^2. **b)** STM image shown in a) with a green lattice corresponding to the moiré periodicity overlaid. In Figure S1b, the non-linear effects inherent to STM scanning at RT have been corrected by using the moiré superstructure as a template.
